# Fine-Grained Semantic Categorization across the Abstract and Concrete Domains

**DOI:** 10.1371/journal.pone.0067090

**Published:** 2013-06-25

**Authors:** Marta Ghio, Matilde Maria Serena Vaghi, Marco Tettamanti

**Affiliations:** 1 Laboratorio di linguistica “G. Nencioni”, Scuola Normale Superiore, Pisa, Italy; 2 Faculty of Psychology, Vita-Salute San Raffaele University, Milano, Italy; 3 Department of Nuclear Medicine and Division of Neuroscience, San Raffaele Scientific Institute, Milano, Italy; University Of Cambridge, United Kingdom

## Abstract

A consolidated approach to the study of the mental representation of word meanings has consisted in contrasting different domains of knowledge, broadly reflecting the abstract-concrete dichotomy. More fine-grained semantic distinctions have emerged in neuropsychological and cognitive neuroscience work, reflecting semantic category specificity, but almost exclusively within the concrete domain. Theoretical advances, particularly within the area of embodied cognition, have more recently put forward the idea that distributed neural representations tied to the kinds of experience maintained with the concepts' referents might distinguish conceptual meanings with a high degree of specificity, including those within the abstract domain. Here we report the results of two psycholinguistic rating studies incorporating such theoretical advances with two main objectives: first, to provide empirical evidence of fine-grained distinctions within both the abstract and the concrete semantic domains with respect to relevant psycholinguistic dimensions; second, to develop a carefully controlled linguistic stimulus set that may be used for auditory as well as visual neuroimaging studies focusing on the parametrization of the semantic space beyond the abstract-concrete dichotomy. Ninety-six participants rated a set of 210 sentences across pre-selected concrete (mouth, hand, or leg action-related) and abstract (mental state-, emotion-, mathematics-related) categories, with respect either to different semantic domain-related scales (rating study 1), or to concreteness, familiarity, and context availability (rating study 2). Inferential statistics and correspondence analyses highlighted distinguishing semantic and psycholinguistic traits for each of the pre-selected categories, indicating that a simple abstract-concrete dichotomy is not sufficient to account for the entire semantic variability within either domains.

## Introduction

Classification in science is crucial. One of the first brilliant examples of it can be found in the work of the Swedish botanist Carl Linnaeus who implemented a naming system for animal and plant organisms that proved to be an elegant solution for the taxonomic literature [1]. Maybe the ultimate goal of a good system of classification is to allow the general knowledge of a given phenomenon to go a step further, certainly not classification per se. Even in the research concerning how meaning is represented in the speaker's mind/brain, classification is not a minor detail. A pivotal categorization is the one between concrete (e.g., banana, hand, table, bolt), and abstract (e.g., peace, love, justice, ideal) meanings, respectively defined as referring to something that can either be directly experienced or not through the senses [2]. Over the last forty years, the dichotomy between concrete and abstract semantic categories has been suggested by data from: (i) rating studies, describing concrete words as more imageable, easier to think of a specific context for, more familiar, and acquired earlier during infancy than abstract words [3]–[5]; (ii) behavioral experiments, demonstrating a concreteness effect, i.e. a cognitive advantage for concrete over abstract meanings in terms of speed and accuracy with which words are processed ([6]–[7]; but see [8]); (iii) neuropsychological research, reporting double dissociations, i.e. cases of patients more impaired with concrete words, as opposed to other patients more impaired with abstract words [9]; (iv) neuroimaging studies, suggesting different neural networks supporting abstract and concrete meaning processing (for reviews, see [10]–[11]). At the theoretical level, the differences between concrete and abstract concepts have been explained in terms of greater availability either of both the perceptual and verbal information [12], or of related contextual information [13] for concrete versus abstract concepts. Concrete concepts were also described as being characterized by a higher number of semantic features [14]. In contrast to such quantitative accounts, according to which abstract and concrete words differ in terms of the amount of information involved, a recent account rather posited qualitative differences between concrete and abstract words. This kind of alternative theoretical proposal was based on evidence collected in patients [15] and crucially also in healthy subjects [16]. Accordingly, it has been suggested that the distinction between concrete and abstract words is embedded in qualitatively different principles of organization for concrete and abstract words, that is, respectively, a categorical versus an associative organization [16].

A limitation of the majority of the aforementioned theoretical accounts on the differences between concrete and abstract meanings is that they do not seem to provide interpretations for subtler sub-categorizations within the concrete and abstract domains. As a matter of fact, beside the more general classification between abstract and concrete meanings, it is also possible to augment the level of categorical resolution both within the concrete and the abstract semantic domains. Within the concrete domain, different categories have been identified. As suggested by Wiemer-Hastings and colleagues [17], concrete items are characterized by salient dimensions that allow them to be readily classified into categories. For example, given a set of concrete words such as *apple*, *cabbage*, *squirrel*, and *duck*, their sorting into different classes, i.e. vegetables and animals, is straightforward. A potential explanation of this phenomenon is that concrete words belonging to the same category would typically share some features, making them more similar to each other than to other items belonging to distinct categories [17]. For example, considering the category of animals, some features such as ‘has ears’ and ‘has a tail’ are shared by many members of the same category [18]. The distinction of concrete meanings into different sub-categories is also supported by neuropsychological and neuroimaging evidence. Brain damaged patients can show deficits restricted to a single domain (e.g., living things, non-living things), or a category (e.g., animals, fruits, tools, musical instruments, body parts) of knowledge [19]. Neuroimaging studies reported sensory modality-specific brain activations for linguistic items referring to entities experienced through senses, such as tactile- [20], taste- [21], sound- [22], odor- [23], and visual-related meanings [24]. The available literature consistently showed that also action-related concepts identify a category with specific neural substrates [25], and whose existence can be inferred by means of behavioral experiments [26]–[27]. Previous neuroimaging studies [28]–[30] also proved that different sub-categories of action-related meanings (such as mouth-, hand-, or leg-related utterances) were somatotopically represented in the left motor and premotor cortex.

The strong overlap between the neural correlates involved in processing semantic knowledge referring to either sensory or motor entities and the neural systems devoted to the sensory-motor experience with those entities, has been formalized particularly over the last fifteen years into the theoretical framework of embodied cognition [31]. Within this framework, the fine-grain distinction between different categories of concrete concepts naturally follows from the general idea that concepts referring to either sensory or motor entities are stored at least in part in the specific neural systems that mediate the experience with the concepts' referents [32]–[33].

What about abstract meanings, then? Is it possible to draw fine-grained categorical distinctions within the abstract domain, similarly as for the concrete domain of conceptual knowledge? Embodied cognition accounts have postulated that also in the abstract domain, the storage of conceptual knowledge may reflect the type of experience that is characteristic for the concepts' referents, with for example an involvement of the neural systems processing emotions for affective concepts, and of the mentalizing neural network for introspective concepts referring to mental states [34]–[35].

Evidence compatible with such a generalized embodied account has more recently begun to emerge (e.g., [36]–[37]), but otherwise the domain of abstract meanings has been scarcely explored and generally regarded as an undifferentiated whole in experimental studies (for a review, see [10]). To start with, the definition of abstract words do not fully characterize abstract concepts, as they are mainly defined by exclusion [38], namely as referring to entities that are neither physically nor spatially constrained. It has also been suggested that, in sharp contrast with concrete words in which features are shared within the same category, categories of abstract items have a low inter-category distinctiveness [17]. For example, similarity ratings for a pair of items belonging to the same abstract category (e.g., events) were lower than similarity ratings for a pair of items belonging to the same concrete category (e.g., plants) [17]. As a consequence, “abstract” has been often used as a wide label including words that do not have physical referents, such as *happiness*, *justice*, and *doubt*, without considering the heterogeneity of this class of meanings [39].

Only few studies have shed light on whether there exist differences between categories of abstract-related concepts. Setti and Caramelli [40] investigated three sub-categories of abstract concepts largely related to mental states (nominal kind, state of the self, and cognitive processes), reporting that each semantic domain showed a specific pattern in concreteness/abstractness and imagery ratings, and a specific pattern of information (taxonomic, thematic, and attributive) in a definition production task.

Another semantic category which has generally been confounded among other instances of the generic abstract category is represented by emotion-related concepts. In a rating study, Altarriba and colleagues [41] showed that, when treated as a separate category, emotion words (e.g., *excited*, *lonely*, *infatuated*, *upset*) were less concrete and lower in context availability, but more imageable than abstract words (e.g., *easy*, *donor*, *travel*, *finish*). In a subsequent memory recall study, the same authors found that emotion words were better remembered than either concrete or abstract words [42], thus revealing the distinctiveness of emotion meanings in comparison to both concrete and abstract meanings. Kousta and colleagues [43] showed in a lexical decision task that, irrespective of valence (namely, positive or negative), emotional words were processed more quickly than neutral words. However, evidence is still not clear cut. For example, in terms of reaction times, either a disadvantage [44]–[45] or an advantage [46] was found for negative emotion words. These controversial results could have been due to different task demands that may modulate the effect of emotions, different criteria for item selection, or sampling differences for valence [43], [8].

As still another potential abstract semantic category, recent studies focused on mathematics-related concepts, considering them as a special case of abstract concepts, with a strong link between numerical representations and the hand fingers used for counting [47]–[49].

This brief review of the specialistic literature clearly indicates that evidence on abstract meanings representation and processing is highly fragmentary, and still limited to restricted lexical-semantic domains. In the present study, we propose that in order to improve our understanding of the processing and representation of the abstract conceptual-semantic domain, the time is ripe for developing a more fine-grained classification. As a first step in this direction, considering previous language studies suggesting the existence of different types of abstract meanings, we putatively distinguished between three different categories within the abstract domain: mental state-related meanings, emotion-related meanings, and mathematics-related meanings. Instead of single words as in most previous studies, we used sentences, which, as we will argue, allow for the resolution of many lexical-semantic confounding side-effects.

Mental state-related meanings mainly referred to several cognitive states expressed by mental state verbs [50] and dealing with abstract entities (e.g., *She contemplates the alternative*).

With respect to emotion-related meanings, differently from most studies aimed at investigating the relationship between language and emotions, we considered only utterances referring to emotions and feelings per se (e.g., *She feels disgust*). We in turn excluded highly arousing utterances referring to actions or entities with an emotional connotation (e.g., *She stabs her husband*; see also [37] proposing a similar approach).

Mathematics-related concepts, as a special case of abstract knowledge with sensory-motor grounding in hand finger representations, referred to calculations and other mathematical operations (e.g., *She counts the sets*).

We compared mental state-, emotion-, and mathematics-related meanings to three action-related meaning categories within the concrete semantic domain. Based on their relevance for evidence-based sensory-motor embodiment, we distinguished between mouth-related (e.g., *She inflates the balloon*), hand-related (e.g., *She plucks the strings*), and leg-related meanings (e.g., *She bends the knee*), since a fine-grained characterization of effector-specific action-related meanings in psycholinguistic terms is still missing.

The first objective of this study was to provide empirical evidence of fine-grained distinctions within both the abstract and the concrete semantic domains with respect to relevant psycholinguistic dimensions. As we suggested above (see also [51]), the abstract and concrete categories are very heterogeneous, including several different classes of meanings that deserve a thorough psycholinguistic and neuroscientific characterization. In the present study, we start by characterizing meanings with respect to several psycholinguistic dimensions, in order to provide psycholinguistic measures that may guide the selection of stimuli in future studies. In line with this, the second aim of this study was to develop a carefully controlled linguistic stimulus set that may be used for auditory as well as visual neuroimaging studies focusing on the parametrization of the semantic space beyond the abstract-concrete dichotomy.

For these purposes, we created a set of Italian sentences that refer to the six semantic classes described above, and carefully controlled for: (i) psycholinguistic characteristics, such as sentence length, lexical frequency, and syntactic form. The effects of these psycholinguistic variables on behavioral responses and brain processes has been clearly demonstrated for linguistic stimuli presented either in the visual or in the auditory modality [52]–[53]; (ii) auditory characteristics, such as prosody, pitch, intensity, and sentence duration, which also influence auditory stimulus processing [54].

Sentences were characterized at the psycholinguistic level by means of two rating studies. Study 1 was aimed at verifying through a rating procedure whether the literature-based distinction of the abstract and concrete domains into different semantic categories was reflected by speaker’s judgments. Participants were asked to evaluate sentences with respect to different semantic domain-related scales, specifically created for measuring if and how sentences were categorized.

In study 2, we measured the concreteness/abstractness of the six semantic categories by means of concreteness ratings. We also characterized the set of stimuli for familiarity and context availability. All these psycholinguistic variables have been used in previous studies to quantify the differences between concrete and abstract meanings at the word level [5], [55]–[56]. The current literature does not provide normative data about concreteness, context availability, or familiarity for sentence stimuli, except for studies considering special types of sentences, such as metaphorical sentences [57]. By collecting these ratings, we aimed at providing standard measures to quantify similarities/dissimilarities among different semantic categories within the concrete and abstract domains, also extending previous results at the sentence level.

This set of stimuli may be used in future neuroscientific and behavioral studies on the processing of different semantic categories either through visual or auditory perception. Relying on the provided rating measures, in future research the factors and psycholinguistic variables considered here (i.e. semantic domains, concreteness/abstractness, length, frequency, familiarity, context availability) may be experimentally manipulated in a factorial or a parametric fashion, either as parameters of interest or as confounds.

## Materials and Methods

### Ethics Statement

All volunteer subjects gave written consent to participate after receiving an explanation of the procedures, according to the Declaration of Helsinki. The study was approved by the Ethics Committee of the San Raffaele Hospital, Milan.

### Linguistic Stimuli

In a series of normative pre-tests, 150 volunteers (different from the ones mentioned below as participants) evaluated different versions of the sentences with respect to different variables. Pre-tests were paper and pencil questionnaires asking participants to judge all sentences on concreteness, context availability, familiarity, and body-part involvement using 7-point Likert scales. Pre-normative results were statistically evaluated in order to guide the final choice of the sentences to be used in the present study.

The 210 selected Italian sentences all consisted of four words and had the same syntactic structure: third person feminine pronoun, verb in third-person singular, simple present tense, matched to a syntactically and semantically congruent object complement. Thirty-five sentences for each of the three abstract-related semantic domains were created: mental state-related sentences (Ms) (e.g., ‘Lei ricorda il passato’, Engl.: *She remembers the past*); emotion-related sentences (Em) (e.g., ‘Lei mostra il disappunto’, Engl.: *She shows her disappointment*); mathematics-related sentences (Ma) (e.g., ‘Lei calcola la somma’, Engl.: *She determines the sum*). Thirty-five sentences for each action-related semantic domain were also formed: mouth-related sentences (Mo) (e.g., ‘Lei schiocca la lingua’, Engl.: *She clicks her tongue*); hand-related sentences (Ha) (e.g., ‘Lei ricama il fazzoletto’, Engl.: *She embroiders the handkerchief*); leg-related sentences (Le) (e.g., ‘Lei calcia la palla’, Engl.: *She kicks the ball*). For simplicity, example sentences in the remainder parts of the paper are only provided in the form of literal English translations from Italian, omitting in turn the original Italian versions.

Experimental stimuli were controlled for length and frequency of use across the six experimental conditions. The length of sentences was measured by the number of words and letters (important if sentences are to be presented in a visual format), and by the number of syllables (important if sentences are to be presented in a spoken format). The frequency of use was controlled by considering two different measures: (i) a measure of lexical frequency of the content words constituting the sentences (e.g., *kicks* and *ball* are the content words of the sentence *She kicks the ball*) on the basis of the available frequency norm of Italian Corpus and Frequency lexicon of written Italian (ColFIS, [58]); (ii) a subjective measure of the sentence frequency was obtained by means of familiarity rating (for details see section *Rating study 1*).

### Linguistic Stimuli in Auditory Form

As this study aimed at providing a set of sentences that can be used in future studies not only in a visual format, but also in an auditory format, we created a recorded version of the set of stimuli as well.

Sentences were pronounced by a female, native speaker of Italian in an anechoic room, while registering in stereo modality with a 96.000 Hz sampling rate and a bit-depth of 16 bit. To avoid prosodic effects, and to minimize possible confounding influences of low-level auditory features such as pitch or accent, all sentences were read with a controlled neutral intonation. After recording, a manipulation procedure was applied to all sentences using Praat 5.2.03 software (www.praat.org, [59]). Praat scripts, available at the Praat Script Archive (www.sites.google.com/site/praatscripts), were specifically modified for: (i) cutting traces, in order to leave no silence at the beginning and at the end of each sentence; (ii) fixing each audio trace to the same amplitude interval (70 dB); (iii) extracting the values of the following parameters: temporal duration, mean intensity and mean pitch.

The complete set of written and auditory Italian sentences and the modified Praat scripts can be obtained by sending requests to M.T. (tettamanti.marco@hsr.it).

### Participants

Ninety-six undergraduate students from the Vita-Salute San Raffaele University, Milan (63 males, mean age = 20.0±0.7) participated to this study. Half of the participants were randomly assigned to group 1 and performed rating study 1, the other half were assigned to group 2 and performed rating study 2. All subjects were native Italian speakers. Education level was highly matched as all participants were attending the first year Medicine course (years of education mean = 13.5±1.5). They were not paid nor received extra credits for their participation. Participants were unaware of the aim of the study, and they were not experts in linguistics nor in the specialistic psycholinguistic and cognitive neuroscientific literature.

### Rating Study 1

Rating study 1 aimed at validating the putative distinction of sentences into six different semantic categories suggested on the basis of the current literature by means of association and body-part ratings.

Association task: for Ms, Em, and Ma sentences, we asked participants to evaluate how much the meaning of each sentence was associated to the meaning of three other sentences (one Ms, one Em, and one Ma) randomly selected from the pool of abstract-related sentences. For example, subjects had to judge how much the meaning of a target sentence like *She feels happy* (Em) was associated to the meaning of the three following sentences: *She memorizes the procedure* (Ms), *She conceals the anger* (Em), and *She calculates the sum* (Ma). For each target sentence, we created a specific triplet in order to use each Ms, Em, and Ma sentence only once; the order of the presentation of the sentences in the triplet was randomized. For each association, a 7-point Likert scale was employed ranging from 1 = “not associated” to 7 = “highly associated”. By way of this association task, we investigated whether different semantic classes could emerge from the rating data, without imposing a priori the semantic categories to which they possibly belonged. More specifically, we expected that Ms, Em, and Ma sentences clustered with their corresponding counterparts.

Body-part task: for Mo, Ha, and Le sentences we asked participants to evaluate how much the action described in each sentence involved the mouth, the hand, and the leg using three body-part Likert scales (mouth scale, hand scale, leg scale) ranging from 1 = “not involved” to 7 = “highly involved” [28], [60]. To better characterize a potential motor dimension of abstract-related sentences, we asked participants to also rate Ms, Em, and Ma sentences.

For both the association and the body-part tasks, two sentence-response examples were provided for reference with the task instructions, using different stimuli than those from the experimental set.

### Procedure Rating Study 1

The pool of 210 sentences was divided into six separate lists. Lists were rotated among the two tasks, i.e. the association task and the body-part task. Five of the lists included 18 target sentences (3 sentences for each of the 6 experimental conditions) for the association rating, and 36 sentences (6 sentences for each of the 6 experimental conditions) for the body-part rating; one list included 15 target sentences for the association rating and 30 sentences for the body-part rating. By means of this procedure, all sentences were scored, avoiding the same subject to rate the same sentence more than once. At the same time, the use of relatively short lists was aimed at preserving a high level of attention throughout the study, and preventing from fatigue. Between lists, the order of the presentation of the tasks was counterbalanced across participants. Within each list, the order of sentences was pseudo-randomized. For each rating, each sentence was rated by 8 participants.

The rating was conducted through a web-based procedure using Survey Monkey (SurveyMonkey.com, LCC, Palo Alto, California, USA, www.surveymonkey.com). Each participant completed the rating study individually on a computer console. Sentences were presented one by one on the screen, and subjects expressed their judgments by clicking on the chosen value of the Likert scales reported under each sentence. This procedure was intended at having a better control over the presentation of items as they were administered in conformity with the sequential order decided by the experimenter. Moreover, participants’ rating scores were directly coded on an Excel database file, avoiding mistakes related to the recording of scores. All consent information and instructions for the tasks were provided in Italian, through the same web-based utility. Altogether, the experimental session took no longer than 20 minutes for each subject.

### Rating Study 2

To quantify and measure the differences between semantic categories, we designed a second rating study in which sentences were rated on concreteness (CNC), context availability (CA), and familiarity (FAM) by means of 7-point Likert scales. The instructions for the concreteness, the context availability, and the familiarity tasks were largely based on those used by previous investigators for single words ([61]; see [56] for the Italian version of the tasks’ instructions), and adapted for use with sentences.

Concreteness task: participants were asked to judge whether the semantic meaning depicted by the sentence either referred to a non-physical situation/state or to a physical action involving objects, materials and/or people (1 = “abstract”, 7 = “concrete”).

Context availability task: subjects were asked to rate the ease with which they could think of a specific context or circumstances associated with the sentence or in which the sentence could appear (1 = “very difficult”, 7 = “very easy”).

Familiarity task: participants judged how often they usually listened to or produced each sentence (1 = “unfamiliar”, 7 = “very familiar”).

A few sentence-response examples were provided for reference with the task instructions, using different stimuli than those from the experimental set.

### Procedure Rating Study 2

Similarly to rating study 1, six lists were created, and rotated among the CNC, CA, and FAM scales so that all sentences were rated on all dimensions but the same subject did not rate the same sentence more than once. An equal number of Ms, Em, Ma, Mo, Ha, and Le sentences were included in each list (3 lists included a total number of 102 sentences, and 3 lists included a total number of 108 sentences). The same procedure of counterbalancing the order of presentation of the rating scales across participants and presenting sentences in a pseudo-randomized order as in rating study 1 was used. Data were collected with the same web-based procedure described for rating study 1.

### Data Analysis

Likert scores obtained in rating study 1 and 2 were analyzed using SPSS 13.0 software (IBM, Somers, NY, USA) and R 2.13.0 [62]. Missing responses (0.06%) in the questionnaires were treated as missing data in the analysis.

There is disagreement between scholars about whether Likert data should be analyzed with a parametric statistics (“liberal” approach) or nonparametric statistics (“conservative” approach) [63]–[66]. A recent study comparing type I and II error rates of a parametric t-test vs. nonparametric Mann Whitney-Wilcoxon test for Likert data [67] showed that both tests generally have equivalent power, except for skewed and peaked distributions for which nonparametric test is superior. Nanna and Sawilowsky [68] found that the Mann-Whitney-Wilcoxon test was superior in all investigated cases of seven-point Likert data which allows for longer tails and more skewness than five-point data. Leys and Schumann [69] also showed that nonparametric tests are more powerful when assumptions underlying the use of parametric tests are violated. For each rating, we analyzed the distribution of Likert data showing that the assumption of normality of data distribution was never verified, and some distributions (e.g., concreteness and leg scales) were skewed. Consequently, for each rating, Likert data were analyzed by applying the following procedure: (i) as far as descriptive statistics is concerned, we used median as a measure of central tendency and inter-quartile range as a measure of dispersion. However, given that the largest majority of literature articles report means and standard deviations for descriptive purposes, we also reported these values to facilitate comparisons with previous studies; (ii) we applied the nonparametric Kruskall-Wallis test on raw data to assess differences in mean ranks across the six experimental conditions; (iii) we used post-hoc Mann–Whitney U tests with Bonferroni correction for multiple comparisons. To further control the results obtained following this procedure, for each rating scale we also conducted parametric analyses, both by items and by subjects, by applying the Univariate General Linear Model. In all cases, the results confirmed those obtained with the non-parametric procedure described above, and are not reported in the Results section.

In addition, in rating study 2, in order to find the latent patterns underlying our stimuli, CNC, CA, and FAM ratings were explored in R statistical software using the “languageR” package [70]–[71] by means of correspondence analysis, an exploratory data technique used to analyze categorical data [72]. The correspondence analysis provides an informative and concise means of visualizing data and it is capable of uncovering relationships both among and between variables. In statistical terms, it tests the association between two variables tallied in the form of a contingency table; graphically, it enables a low dimensional configuration of the associations between the rows and the columns of the contingency table. The goals of the correspondence analysis are to reduce the dimension original space, and to find an optimal subspace that is closest to the cloud of points in the chi square-metric. The loss of information associated with this dimension reduction is quantified in terms of the proportion of the so-called inertia that is explained by the axes displayed. To decide how many dimensions (hereafter named as “factors” according to [71]) are needed to explain the variation in the data we used the screeplot, in which the factors' eigenvalues are plotted in order of magnitude from largest to smallest. An “elbow” in the plot, that is a change in slope in the diagram, corresponds to the point where there is a marked drop in the amount of variation explained. Factors with inertia contribution higher than this elbow were selected for interpretation, whereas the factors forming the elbow or lower than the elbow were not further considered. The coordinates of both row and column points of the chi-square contingency table were projected onto the selected low-dimensional subspace: in this representation, row and column points that are close together are more alike than points that are far apart. Finally, in order to describe the distribution of points with respect to the six semantic categories, for each factor we plotted the mean coordinates of the points of each category by means of barplots. These mean coordinates were also statistically compared with respect to the six semantic categories.

Non-parametric Spearman’s rank-order correlations (r_s_) were calculated in order to assess the relations among: (i) CNC, CA, FAM ratings with respect to all sentence categories; (ii) CNC and body-part ratings with respect to abstract-related categories.

## Results

### Linguistic and Auditory Characteristics

Linguistic and auditory characteristics are shown in Table 1. Nouns and verbs frequency were balanced across the six semantic categories (nouns: F(5,204) = 1.861; p = 0.103; verbs: F(5,204) = 1.723; p = 0.131; noun-verb combinations: F(5,204) = 1.824; p = 0.110). The length of the stimuli was also controlled: all sentences had four words and the number of letters was balanced across categories (F(5,204) = 1.250; p = 0.287). However, when considering the number of syllables, we found a trend toward a main effect of the semantic category (χ^2^(25) = 36.371; p = 0.066).

**Table 1:** Descriptive statistics of linguistic and auditory characteristics for (Ms) mental state-,(Em) emotion-, (Ma) mathematics-, (Mo) mouth-, (Ha) hand-, and (Le) leg-related sentences.

	No. ofwords	No. ofsyllables	No. ofletters	Frequencyverb	Frequencynoun	Frequencyverb+noun	Intensity(dB)	Pitch(Hz)	Duration(sec)
	Mean (SD)	Mean (SD)	Mean (SD)	Mean (SD)	Mean (SD)	Mean (SD)	Mean (SD)	Mean (SD)	Mean (SD)
**Ms**	4.00	8.14	19.66	45.96	50.56	96.52	70.09	232.85	1.47
	(.00)	(.88)	(1.86)	(59.82)	(47.61)	(67.87)	(0.02)	(4.26)	(0.11)
**Em**	4.00	7.63	19.03	61.43	30.01	91.44	70.07	230.41	1.44
	(.00)	(1.11)	(2.67)	(82.57)	(46.34)	(88.69)	(0.04)	(6.03)	(0.17)
**Ma**	4.00	8.00	19.29	57.07	56.25	113.32	70.07	232.09	1.44
	(.00)	(.69)	(1.43)	(93.17)	(90.27)	(139.53)	(0.02)	(5.12)	(0.12)
**Mo**	4.00	7.37	18.91	12.05	32.15	44.20	70.08	230.59	1.39
	(.00)	(1.09)	(2.85)	(34.84)	(52.22)	(65.77)	(0.03)	(4.52)	(0.15)
**Ha**	4.00	7.49	18.43	46.70	26.50	73.20	70.07	231.75	1.38
	(.00)	(.82)	(2.23)	(131.54)	(38.35)	(136.25)	(0.02)	(4.40)	(0.13)
**Le**	4.00	7.43	18.71	66.37	27.29	93.66	70.07	230.81	1.40
	(.00)	(.88)	(2.35)	(93.20)	(44.78)	(104.40)	(0.03)	(4.03)	(0.12)

Statistical analysis of auditory features revealed that mean intensity (F(5,204) = 1.465; p = 0.203), and mean pitch (F(5,204) = 1.433; p = 0.214) of sentences were balanced across the six semantic categories. We found that the difference of sentence duration across categories reached the threshold of significance (F(5,204) = 2.259; p = 0.050).

### Study 1: Association Rating

Table 2 presents descriptive statistics (median, inter-quartile range, mean, standard deviation) showing how Ms, Em, and Ma sentences were associated to the meaning of sentences belonging, respectively, to the mental-state, emotion, and mathematics-related semantic domain.

**Table 2:** Descriptive statistics of association ratings for (Ms) mental state-, (Em) emotion-, (Ma) mathematics-related sentences.

	Mental-state association scale		Emotion association scale		Mathematics association scale	
	Mdn (IQR)	Mean (SD)	Mdn (IQR)	Mean (SD)	Mdn (IQR)	Mean (SD)
**Ms** (n = 35)	5 (3–6)	4.31 (2.31)	2 (1–3)	2.38 (1.77)	1 (1–4)	2.4 (1.97)
**Em** (n = 35)	2 (1–3)	2.34 (1.73)	5 (3–5)	4.75 (1.98)	1 (1–1)	1.46 (1.19)
**Ma** (n = 35)	1 (1–4)	2.41 (1.82)	1 (1–1)	1.53 (1.27)	6 (4–7)	5.23 (1.86)

**Figure 1:**
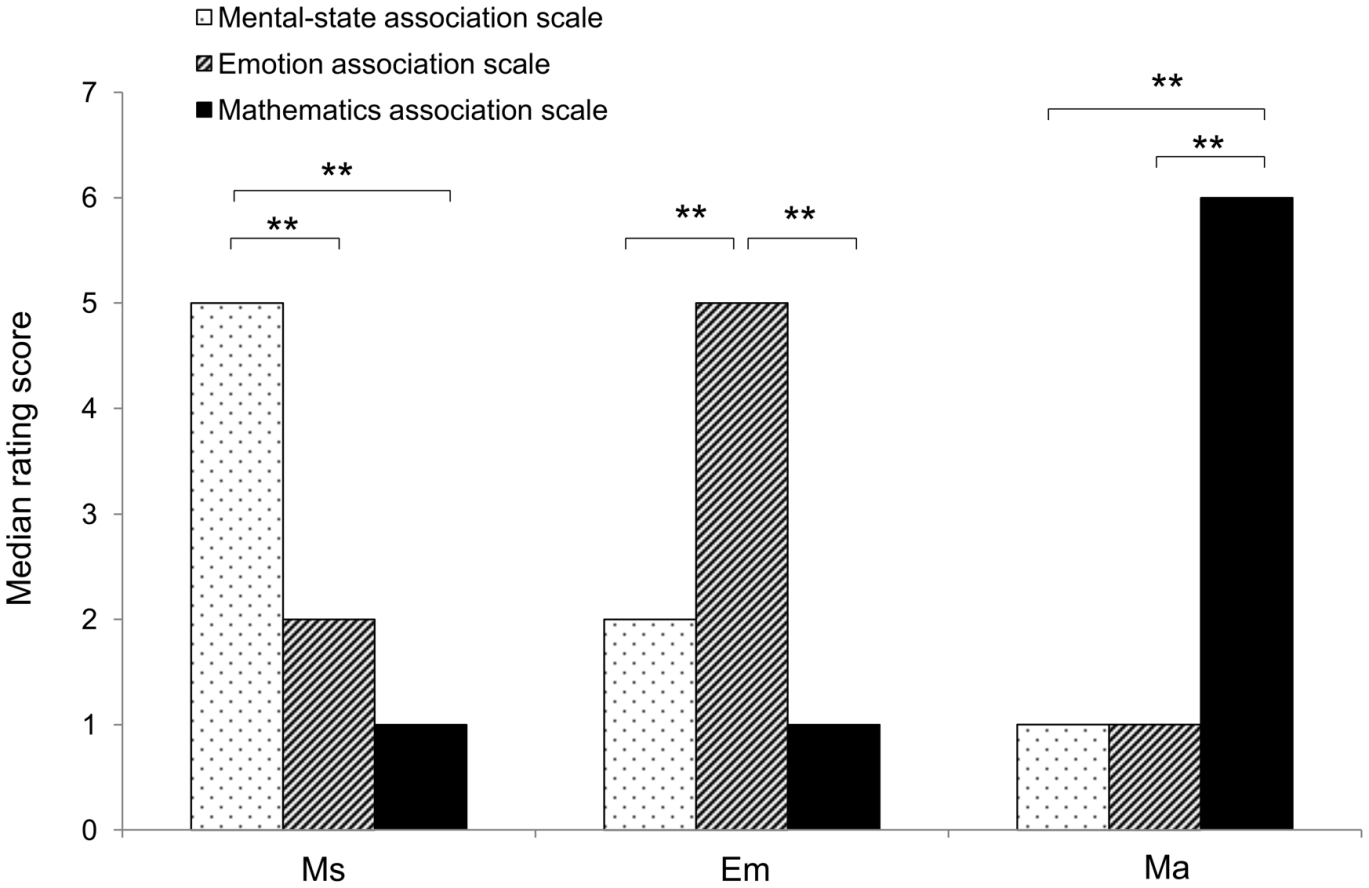
Association rating scores. Bar plot showing median association rating scores for (Ms) mental state-, (Em) emotion-, and (Ma) mathematics-related sentences (*p<0.05, **p<0.01).

We found a significant effect of the semantic domain for each group of abstract-related sentences (Figure 1). Specifically, Ms sentences received higher scores for the mental-state association scale than for the two other scales (χ^2^(2) = 148.484; p<0.001; Mann Whitney pairwise comparisons, all p<0.001); Em sentences received higher scores for the emotion association scale than for the two other scales (χ^2^(2) = 360.371; p<0.001; Mann Whitney pairwise comparisons, all p<0.001); Ma sentences received higher scores for the mathematics association scale than for the two other scales (χ^2^(2) = 381.572; p<0.001; Mann Whitney pairwise comparisons, all p<0.001). To exclude similarities across different semantic domains, for each association scale we compared the median association scores obtained by the sentences belonging to the three different semantic domains (Figure 1). We found that Ms sentences were significantly more associated with Ms sentences than were Em and Ma sentences (χ^2^(2) = 151.455; p<0.001; Mann Whitney pairwise comparisons, all p<0.001); Em sentences were significantly more associated with Em sentences than were Ms and Ma sentences (χ^2^(2) = 342.740; p<0.001; Mann Whitney pairwise comparisons, all p<0.001); Ma sentences were significantly more associated with Ma sentences than were Ms and Em sentences (χ^2^(2) = 381.909; p<0.001; Mann Whitney pairwise comparisons, all p<0.001).

### Study 1: Body-part Rating

Table 3 presents descriptive statistics (median, inter-quartile range, mean, standard deviation) describing how each group of sentences was judged for the three action-related scales.

**Table 3:** Descriptive statistics of body-part ratings for (Mo) mouth-, (Ha) hand-, (Le) leg-, (Ms) mental state-, (Em) emotion-, (Ma) mathematics-related sentences.

	Mouth scale		Hand scale		Leg scale	
	Mdn (IQR)	Mean (SD)	Mdn (IQR)	Mean (SD)	Mdn (IQR)	Mean (SD)
**Mo** (n = 35)	7 (7–7)	6.81 (0.57)	2 (1–4)	2.88 (1.87)	1 (1–1)	1.13 (0.49)
**Ha** (n = 35)	1 (1–2)	1.49 (1.04)	7 (7–7)	6.61 (0.87)	1 (1–2)	1.50 (1.13)
**Le** (n = 35)	1 (1–1)	1.41 (0.98)	3 (1–4)	2.93 (1.92)	7 (7–7)	6.59 (1.05)
**Ms** (n = 35)	2 (1–5)	3.15 (2.23)	1 (1–3)	2.21 (1.74)	1 (1–1)	1.30 (0.93)
**Em** (n = 35)	4 (1–6)	3.84 (2.34)	2 (1–5)	2.93 (2.17)	1 (1–3)	2.06 (1.74)
**Ma** (n = 35)	1 (1–3)	2.24 (1.70)	3 (1–5)	3.06 (1.90)	1 (1–1)	1.11 (0.59)

**Figure 2:**
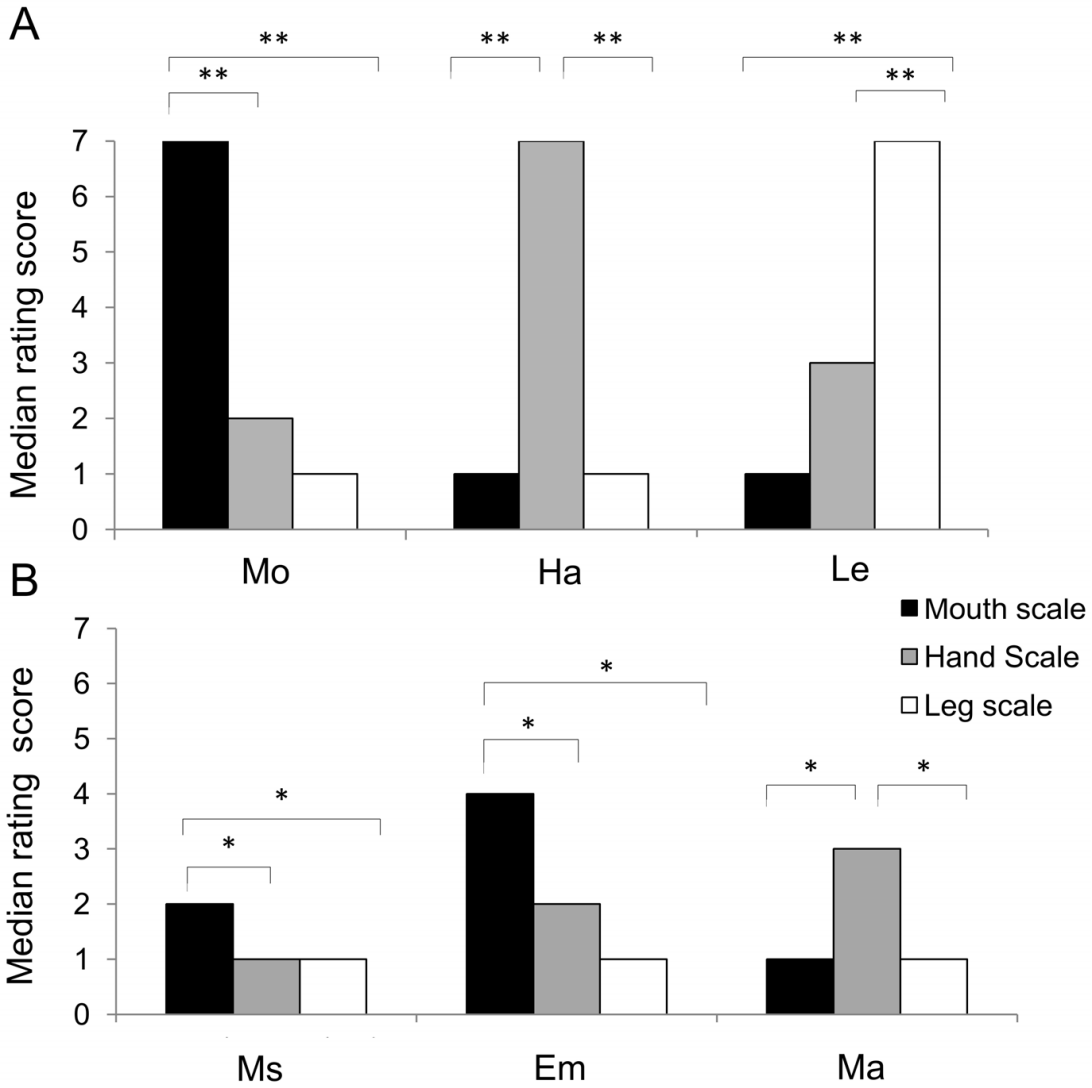
Body-part rating scores. Bar plots showing median body-part rating scores for: A) (Mo) mouth-, (Ha) hand-, and (Le) leg-related sentences, and B) (Ms) mental state-, (Em) emotion-, and (Ma) mathematics-related sentences (*p<0.05, **p<0.01).

For action-related sentences, we found that the three groups of sentences were different from each other, and also significantly different from abstract-related sentences (Figure 2). Specifically, actions described by Mo sentences were judged as involving the mouth significantly more than the hands or the legs (χ^2^(2) = 665.939; p<0.001; Mann Whitney pairwise comparisons, all p<0.001); actions described by Ha sentences were judged as involving hands significantly more than the mouth or the legs (χ^2^(2) = 608.299; p<0.001; Mann Whitney pairwise comparisons, all p<0.001); actions described by Le sentences were judged as involving the legs significantly more than the mouth or the hands (χ^2^(2) = 568.916; p<0.001; Mann Whitney pairwise comparisons, all p<0.001).

For each body-part scale, we also verified the hypothesis of an association between each group of action-related sentences and the specific effector involved (Figure 2A). Ratings for the mouth scale revealed that Mo sentences were significantly more associated with the mouth than were Ha, Le, Ms, Em, and Ma sentences (χ^2^(5) = 848.326; p<0.001; Mann Whitney pairwise comparisons, all p<0.001). Considering the hand scale, Ha sentences were significantly more associated with the hands than were Mo, Le, Ms, Em and Ma sentences (χ^2^(5) = 607.613; p<0.001; Mann Whitney pairwise comparisons, all p<0.001). Consistently, Le sentences were judged as significantly more associated with the legs than were Mo, Ha, Ms, Em, and Ma sentences (χ^2^(5) = 1013.41; p<0.001; Mann Whitney pairwise comparisons, all p<0.001).

For abstract-related sentences, results showed that, when explicitly required, subjects judged the content described by Ms, Em, and Ma sentences as significantly involving different effectors (Figure 2B). Specifically, the semantic content of Ms sentences was more associated with mouth actions than with hand or leg actions (χ^2^(2) = 146.577; p<0.001; Mann Whitney pairwise comparisons, all p<0.001). The semantic content of Em sentences was more associated with mouth actions than with hand or leg actions (χ^2^(2) = 88.742; p<0.001; Mann Whitney pairwise comparisons, all p<0.001). Finally, the semantic content of Ma sentences was more associated with hand actions than with mouth or leg actions (χ^2^(2) = 227.500; p<0.001; Mann Whitney pairwise comparisons, all p<0.001). Considering each scale, Mann Whitney pairwise comparisons showed significant differences between Ms, Em, and Ma sentences. Ratings for the mouth scale indicated that Em sentences were significantly more associated to mouth actions than were either Ms and Ma sentences (p = 0.001); moreover Ms sentences received higher median score than Ma sentences (p<0.001). Ratings for the hand scale revealed that Ma sentences and Em sentences were significantly more associated to hand actions than were Ms sentences (all p<0.001). Considering the leg scale, Em sentences were significantly more associated with leg actions than were Ma and Ms sentences (all p<0.001).

### Study 2: Concreteness Rating

**Figure 3:**
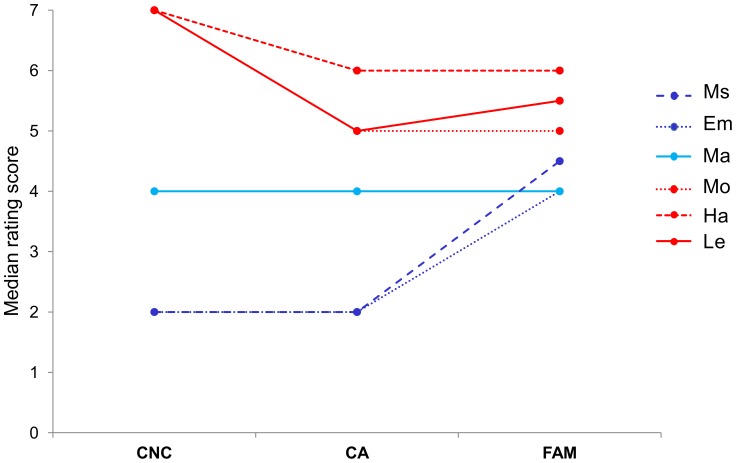
Concreteness, context availability, and familiarity rating scores. Line graph showing median (CNC) concreteness, (CA) context availability, and (FAM) familiarity rating scores for the six categories of sentences. (Ms) mental state-, (Em) emotion-, (Ma) mathematics-, (Mo) mouth-, (Ha) hand-, and (Le) leg-related sentences.

We found a significant effect of the semantic domain (χ^2^(5) = 1117.396; p<0.001). Based on Mann Whitney pairwise comparisons, four significantly different groups were identified: (i) Ms and Em sentences (Ms vs. Em, p = 0.297; all other comparisons: p<0.001); (ii) Ma sentences (all p<0.001); (iii) Mo sentences (all p<0.001); (iv) Ha and Le sentences (Ha vs. Le, p = 0.211; all other comparisons: p<0.001) (Table 4, Figure 3).

**Table 4:** Descriptive statistics of CNC (concreteness), CA (context availability), and FAM (familiarity) ratings for (Ms) mental state-, (Em) emotion-, (Ma) mathematics-, (Ha) hand-, (Le) leg-, and (Mo) mouth-related sentences.

	CNC		CA		FAM	
	Mdn (IQR)	Mean (SD)	Mdn (IQR)	Mean (SD)	Mdn (IQR)	Mean (SD)
**Ms** (n = 35)	2 (1–3)	2.41 (1.52)	2 (1–4)	2.9 (1.9)	4.5 (2–6)	4.19 (1.97)
**Em** (n = 35)	2 (1–3)	2.27 (1.43)	2 (1–4)	2.75 (1.85)	4 (2–6)	4.19 (2.09)
**Ma** (n = 35)	4 (2–5)	3.61 (1.72)	4 (2–6)	3.86 (2.06)	4 (2–6)	3.89 (2.06)
**Mo** (n = 35)	7 (6–7)	6.24 (1.19)	5 (3–6.75)	4.68 (1.97)	5 (4–7)	4.98 (1.89)
**Ha** (n = 35)	7 (7–7)	6.64 (0.8)	6 (4–7)	5.25 (1.79)	6 (4–7)	5.23 (1.97)
**Le** (n = 35)	7 (6–7)	6.52 (1)	5 (3–7)	4.93 (1.90)	5.5 (4–7)	5.04 (1.98)

**Figure 4:**
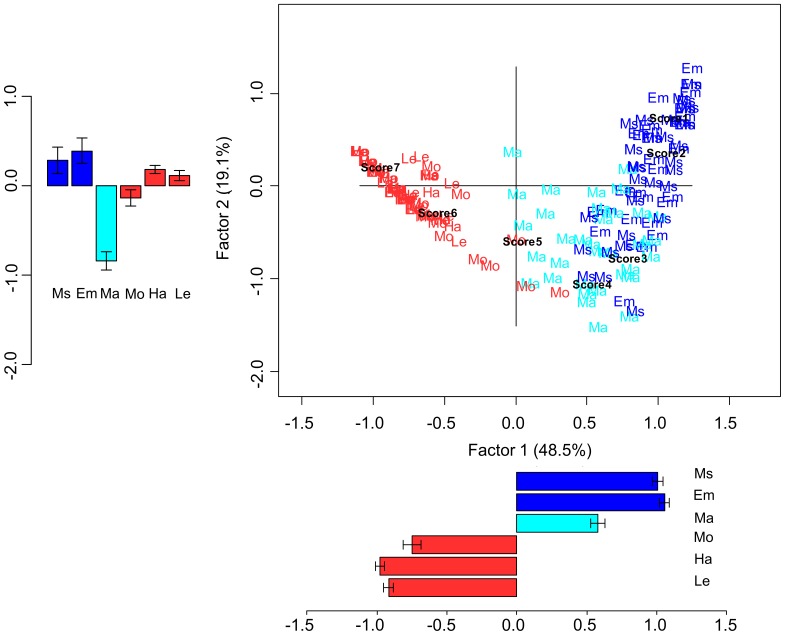
Correspondence analysis for concreteness rating scores. The 210 sentences belonging to the six categories and the 7 Likert points are plotted at their corresponding coordinates. The first and the second factor accounted for the 48.5% and the 19.1% of the total inertia, respectively. Barplots indicate mean coordinates for each factor and category of sentences; error bars indicate standard error means. Action-related (Ha, Mo, Le) sentences are shown in red. Abstract-related sentences are displayed in blue (Ms,Em) and cyan (Ma).

A correspondence analysis was performed with the 210 sentences as one variable (35 Ms, 35 Em, 35 Ma, 35 Mo, 35 Ha, 35 Le) and Likert scores as the other variable. The Chi-square test was significant (χ^2^(1254) = 2624.613; p<0.001), indicating an association between variables. The resulting scree plot revealed a marked decrease in the proportion of inertia explained by the third and subsequent eigenvalues, thus suggesting that a two-factor solution comprising only the first and second factors provided a parsimonious decomposition of the original data. The first and the second factors accounted for 48.5% and 19.1% of the total inertia, respectively. As shown in Figure 4, the first factor roughly separated Mo, Ha, and Le from Ms, Em, and Ma sentences, and may be interpreted to reflect the abstract-concrete dichotomy. By statistically comparing the coordinates along the first factor with respect to sentence categories, a significant difference was found between action-related and abstract-related sentences (t(208) = −41.405; p<0.001). Considering the first factor with respect to the Likert scores, we observed that it was organized according to the exact order of the Likert scale values, with 7 as the leftmost score on the plot and subsequent scores in decreasing order taking a more and more rightward position (Figure 4). As for the second factor, we observed a separation between Ma sentences on the one side and Ms and Em sentences on the other side, thus highlighting a dissociation within the abstract domain. By statistically comparing the coordinates along the second factor, a significant difference was found between Ms and Em vs. Ma (t(88.097) = 8.057; p<0.001). Moreover, the coordinates of Ma were significantly different from those of action-related sentences (t(44.853) = 7.854; p<0.001).

### Study 2: Context Availability Rating

A significant effect of semantic domain was found (χ^2^(5) = 345.279; p<0.001). Mann Whitney pairwise comparisons revealed significant differences between the following subgroups: (i) Ms and Em sentences (Ms vs. Em, p = 0.327; all other comparisons: p<0.001); (ii) Ma sentences (all p<0.001); (iii) Mo and Le sentences (Mo vs. Le, p = 0.120; all other comparisons: p<0.001); (iv) Le and Ha sentences (Ha vs. Le, p = 0.057; all other comparisons: p<0.001) (Table 4, Figure 3).

**Figure 5:**
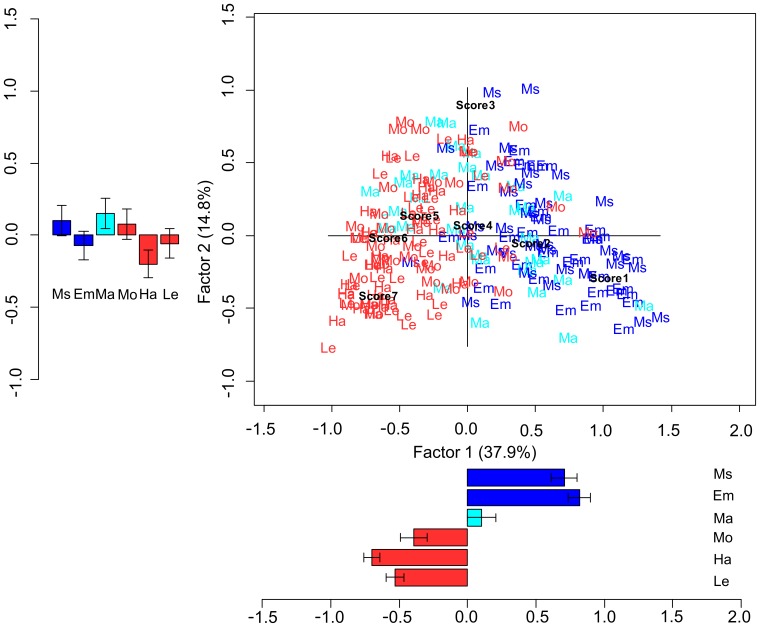
Correspondence analysis for context availability rating scores. The 210 sentences belonging to the six categories and the 7 Likert points are plotted at their corresponding coordinates. The first and the second factor accounted for the 37.9% and the 14.8% of the total inertia, respectively. Barplots indicate mean coordinates for each factor and category of sentences; error bars indicate standard error means. Action-related (Ha, Mo, Le) sentences are shown in red. Abstract-related sentences are displayed in blue (Em, Ms) and cyan (Ma).

The correspondence analysis revealed an association between the sentences belonging to the six semantic categories and CA Likert scores (χ^2^(1254) = 1576.656; p<0.001). The scree plot indicated a marked decrease in the proportion of inertia explained by the second and subsequent eigenvalues; the second and the following factors were therefore not further considered (for additional confidence, we analyzed the second factor coordinates and did not find any significant effects). The first factor, accounting for 37.9% of the total inertia, roughly separated action-related sentences from Ms and Em sentences, with Ma sentences showing a more dispersed distribution (Figure 5). Factor 1 thus seems to reflect the abstract-concrete dichotomy, but with Ma sentences forming a separate category. By statistically comparing the coordinates along the first factor with respect to sentence categories, we observed a significant difference between: action-related and abstract-related sentences (t(136.562) = −16.962; p<0.001), Ma and abstract-related sentences (t(59.756) = 5.523; p<0.001), and Ma and action-related sentences (t(48.140) = −5.766; p<0.001). As for the Likert scores, the first factor was organized according to the exact order of the Likert scale values, with 7 as the leftmost score on the plot and subsequent scores in decreasing order taking a more and more rightward position (Figure 5).

### Study 2: Familiarity Rating

We found a significant effect of semantic domain (χ^2^(5) = 109.383; p<0.001), with Ms, Em, and Ma sentences judged as significantly less familiar than action-related sentences (Mann Whitney comparisons, all p<0.001). No differences were found neither between abstract-related sentences (all p>0.05; alpha level corrected for multiple comparisons = 0.003) nor action-related sentences (all p>0.04; corrected alpha level = 0.003) (Table 4, Figure 3).

**Figure 6:**
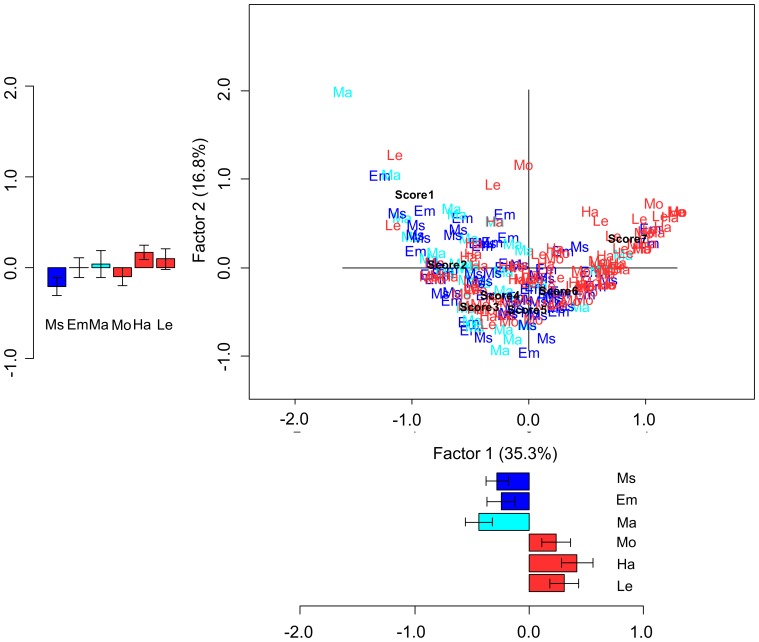
Correspondence analysis for familiarity rating scores. The 210 sentences belonging to the six categories and the 7 Likert points are plotted at their corresponding coordinates. The first and the second factor accounted for the 35.3% and the 16.8% of the total inertia, respectively. Barplots indicate mean coordinates for each factor and category of sentences; error bars indicate standard error means. Action-related (Ha, Mo, Le) sentences are shown in red. Abstract-related sentences are displayed in blue (Ms,Em) and cyan (Ma).

Also for familiarity, an association between the sentences belonging to the six semantic categories and Likert scores was revealed by the correspondence analysis (χ^2^(1254) = 1776.257; p<0.001). The scree plot indicated a marked decrease in the proportion of inertia explained by the second and subsequent eigenvalues; the second and the following factors were therefore not further considered (for additional confidence, we analyzed the second factor coordinates and did not find any significant effects). The first factor, accounting for 35.3% of the total inertia, roughly separated action-related sentences from abstract-related sentences (Figure 6), thus again most likely reflecting the abstract-concrete dichotomy. The distinction of action-related vs. abstract-related sentences into two clusters was confirmed by the analysis of the coordinates along the first factor (t(203.871) = −6.496; p<0.001). To exclude a possible alternative interpretation in terms of lexical frequency instead of familiarity, we compared the coordinates of high vs. low frequency sentences, and no differences were found (t(208) = 1.244; p = 0.215). As for the Likert scores, the first factor was organized according to the exact order of the Likert scale values, from 7 as the leftmost score to 1 as the rightmost score on the plot (Figure 6).

### Correlation Analysis

We calculated the correlations for CNC, CA, FAM variables across semantic categories. Consistently with the extant literature [57], all the variables significantly correlated with each other: CNC and CA (r_s_ = 0.745; p<0.01); CNC and FAM (r_s_ = 0.440; p<0.01); CA and FAM (r_s_ = 0.521; p<0.01).

Following Altarriba et al. [41], we also calculated the relation among variables within each semantic group. We found that CNC and CA did not correlate (Spearman’s correlation on median scores: all p>0.05). CA and FAM were significantly correlated for Ms (r_s_ = 0.568; p<0.001), Em (r_s_ = 0.381; p<0.05), Ma (r_s_ = 0.635; p<0.001), and Mo (r_s_ = 0.449; p<0.001) sentences, but did not correlate for Ha and Le sentences (p>0.05). FAM and CNC were correlated for Em sentences only (r_s_ = 0.456; p<0.001).

Finally, in order to characterize the possible relationship between the rated involvement of the three body parts and the perceived concreteness of each abstract-related category, we also calculated abstract category-specific correlations between CNC and body-part ratings (Table 5). Significant correlations were found between CNC and all three body-part scores for Em sentences, and between CNC and mouth-related scores for Ms sentences.

**Table 5:** Correlations between (CNC) concreteness and Mouth, Hand, and Leg ratings calculated for (Ms) mental state-, (Em) emotion-, (Ma) mathematics-related sentences (Spearman’s rank-order coefficients (r_s_ ) on median value; *p<0.05; **p<0.01).

		Mouth scale	Hand scale	Leg scale
**Ms**	**CNC**	0.385*	0.272	0.211
**Em**	**CNC**	0.426*	0.453**	0.463**
**Ma**	**CNC**	−0.038	0.308	–

### Cross-study Validation

For cross validation purposes, we conducted correlations between our data at sentence-level and relevant word-level normative data publicly available. As our stimuli are in Italian, we referred to data of a norming study on Italian words by Della Rosa et al. [56], which is the yet widest normative study in Italian providing concreteness, context availability, and familiarity scores. In this study [56], nouns were taken from the MRC Psycholinguistic Database [55], translated from English to Italian, and rated for the variables of interest. No verbs were included in [56], so our correlations are limited to the noun grammatical category.

Thirty five out of the total of 210 nouns in our stimulus set were available in Della Rosa et al.’s dataset [56]. Correlations were done on this small subset of stimuli on CNC (r_s_ = 0.815; p<0.001), CA (r_s_ = 0.670; p<0.001), and FAM (r_s_ = 0.195; p = 0.262) scales. As expected, a high level of coherence was found for CNC and CA between the word-level noun ratings of [56] and the corresponding sentence-level ratings collected in our study for sentences containing the same nouns. In turn, we did not find any significant correlations for the FAM scale. This may not be much surprising, since the noun *horse*, for example, may be rated as highly familiar as an isolated word, whereas a sentence including *horse*, such as *She rams the horse*, might have been encountered/used relatively infrequently and thus obtain a low familiarity score.

We believe that, in spite of the limited sample (35 out of 210 cases), the fact that the between-sets correlations for CNC and CA were highly significant allows us to conclude with sufficient confidence that the ratings we have collected at the sentence level do not provide a biased picture with respect to available data at the word level. For further confidence in the generalizability of our between-sets correlation results, we also performed nonparametric bootstrapping simulations [73] in order to estimate the extent to which the results obtained with such a limited sample may still hold in the probabilistic scenario of much larger samplings. We let the R statistical software randomly resample the 35 rating pairs (i.e. the pairs constituted by our ratings and those of Della Rosa et al., [56]) 10′000 times with replacements, and we then calculated the ensuing distribution of the Spearman correlation scores for each simulated sample together with the 95% percentile Confidence Intervals (CI) [74]. The results of the bootstrapping simulations confirmed the high correlation between the word-level noun ratings of Della Rosa et al. [56] and the corresponding sentence level ratings collected in our study for CNC (r_s_ = 0.815; 95% CI = 0.629–0.917) and CA (r_s_ = 0.670; 95% CI = 0.409–0.806), but not for FAM (r_s_ = 0.195; 95% CI = −0.139–0.477).

## Discussion

Until now, psycholinguistic studies investigated semantic knowledge by showing a dichotomy between abstract and concrete meanings [75]. However, there is increasing evidence from neuroimaging studies that the neural networks involved in the representation of meanings are flexible and extended throughout the cerebral cortex [24]–[25], thus suggesting that the simplistic classical dichotomy between abstract and concrete meanings has little explanatory power. Such evidence brings into question theoretical accounts explaining the differences between concrete and abstract concepts, both in terms of quantitative [12]–[13] or qualitative [15]–[16] differences, without considering within-domain distinctions. Furthermore, experimental data are pivotal to grounded theories of semantics, according to which the conceptual representation of a semantic category can be viewed as a collection of the multimodal information that has been experienced and processed for instances of that category [76]–[77]. In general, concrete meanings are thought to mainly rely on modalities and systems that process perception and action, while abstract meanings have been suggested to bear on internal states [32], [34]. Assuming a more specific categorization of the concrete domain, it has been shown that the conceptual-semantic language processing of, for example, utterances whose semantic content is related to a particular sensory modality relies on distributed neural networks including the sensory-motor system [22]–[23], [78]–[79]. Conversely, evidence about the semantic networks supporting the processing of different types of abstract meanings is sparse. One reason may be the under-specification of abstract-related meanings so far. A much finer distinction of subordinate referential domains in the abstract domain is nevertheless possible and should by now be taken into consideration. For instance, the above mentioned “internal states”, considered relevant for abstract-related meanings, include: interoception (e.g., affective valence, arousal, hunger, pain, visceral activity, muscle tension), mentalizing (e.g., self-related thoughts, evaluations, representing the thoughts of others, representing how one is perceived by others), attention, reward, affects, executive processing, memory, and reasoning [76]. All these different internal states could be systematically operationalized at the experimental level in future studies, as done at least in part here.

In this study we have offered a psycholinguistic characterization of different conceptual-semantic categories, with a special focus on abstract-related meanings. These data may be quite helpful for future studies aimed at unraveling the grounding of semantic language processing, mainly for two reasons: i) a more accurate description of the psycholinguistic characteristics of categories within the concrete and abstract domains may provide further hints on the type of information included/aggregated to form a conceptual representation; ii) data about psycholinguistic variables such as length, frequency, concreteness, context availability, familiarity, and body-part involvement can be better controlled, as we will suggest, within a parametric experimental approach. Notably, this stimulus set may be suitable for behavioral and neuroimaging research aimed at investigating semantic processing by means of experimental paradigms employing either visually or auditorily presented linguistic stimuli. Relevant linguistic features, i.e. sentence length and lexical frequency have been controlled for all the sentence categories. Familiarity ratings, considered as a subjective measure of frequency [80], revealed that action-related categories were significantly more familiar than abstract-related categories. In order to extend the range of utilization of these stimuli and to make auditory presentation feasible as well, the digitally recorded sentences were matched for mean intensity, mean pitch, and temporal duration, minimizing the possible influences of low-level auditory features. Indeed, a measurable impact of these linguistic characteristics on language processing has been demonstrated both at the behavioral and neural level not only for words, but also when more complex linguistic structures are used [57]. As a further feature of this stimulus set, syntactic complexity was comparable across sentences, with all sentences having the same phrasal structure (i.e., subject+verb+object). While most of the previous studies investigated concrete/abstract differences at the single words level, here we used sentences, thus contributing to the depiction of domain-specific meanings at the sentence level. The use of single words in the research on conceptual processing could have suffered from some confounding side-effects. It has been shown that processing a single verb requires not only to determine its meaning and its syntactic category, but also to establish what arguments it may or must take and what general types of meanings these arguments must have [81]. For example, Ferretti and colleagues [82] found that verbs immediately prime typical agents and patients, suggesting that readers immediately compute typical entities fitting thematic roles associated with verbs on the basis of their schematic knowledge representations. It has also been observed that many nouns, without an available context, contain elements of vagueness or indeterminacy of their meaning (e.g., ambiguous or polysemous nouns) [83]. These observations suggest that single words, especially verbs (e.g., *to grasp*, *to kick*), if presented in isolation, could trigger different interpretations, ranging from a concrete one (e.g., *to grasp the pen*, *to kick the ball*) to an abstract one (e.g., *to grasp the concept*, up to the idiomatic expression like *to kick the bucket*), thus potentially yielding to an inconsistent classification of experimental stimuli. Providing verbs and nouns within a sentence structure, we linguistically contextualized the meanings thus avoiding also this potential drawbacks.

With our cross-study correlations and bootstrapping simulations, comparing the word-level noun ratings of Della Rosa et al. [56] and the corresponding sentence-level ratings collected in our study for sentences containing the same nouns, we nevertheless controlled that, from a psycholinguistic point of view, the data we have collected at the sentence level do not provide a biased picture with respect to available data at the word level.

In particular, we considered three categories of concrete, action-related meanings, namely mouth-, hand-, and leg-related sentences, and three categories of abstract meanings, namely emotion-, mathematics-, and mental state-related sentences. By this, we aimed to validate by means of psycholinguistic rating methods, a set of semantic domains – particularly the abstract Ms, Em, Ma semantic categories – for which some evidence on their category status was already available in the extant literature. This is obviously not meant to exclude that a number of other relevant categories may be identified in either the concrete and abstract domains, such as, just to mention one, the category of “social concepts” [39].

At a broad level, our results consistently reflected the classical dichotomy between concrete and abstract meanings: action-related sentences resulted as more concrete, easier to think a context for, and more familiar than Ms, Em, and Ma sentences. This is in agreement with the vast literature on concrete and abstract single words [3]–[5], [83], but, importantly, it extends the validity of these findings from single word to sentence processing.

At a finer level, in rating study 1 we showed that abstract sentences were clustered into three groups, demonstrating that different types of abstract-related meanings were identified by language users, even if they were not asked to explicitly distinguish between different categories. Alternatively, the results of rating study 1 may be interpreted as an evidence of sentence clustering based not solely on semantic relatedness, but possibly also on the association strength between lexical items. However, we believe that this does not jeopardize an interpretation of our findings in terms of semantic relatedness, given that associative and semantic relations seem to be intrinsically intertwined. The distinction between association based on lexical co-occurrence and semantic relatedness has been questioned in a number of research studies [51], [84]. Indeed, it seems empirically difficult to consider the net effect of one type of relation after excluding the other one: for instance, McNamara [84] directly challenged anyone to find two highly associated words that are not semantically related in some plausible way. The observation that associatively related words are almost unavoidably semantically related has been empirically corroborated by Brainerd et al. [85], showing a correlation between a number of semantic variables and word association strength. It has been shown that lexical co-occurrence is correlated with associative strength [86] and lexical co-occurrence has been proposed as a less costly and more reliable source of association norms [87]. The dividing line between associative and semantic relatedness is then completely blurred in models of semantic representations based on word co-occurrence over text corpora, such as Latent Semantic Analysis [88] and Hyperspace Analogue to Language [89], in which semantic spaces are derived from co-occurrence statistics. In this sense, the association strength between lexical items of sentences belonging to the same semantic category (e.g., *anger* and *happiness* in Em sentences) may be higher than for lexical items of different semantic categories (e.g., *procedure* in Ms sentences, and *sum* in Ma sentences), as lexical co-occurrence is intrinsically related to meaning aspects.

Moreover, the correspondence analysis of rating study 1 then revealed that the dichotomy between abstract and action-related meanings was not sufficient to account for the total data variability. The category-specific correlation patterns provided further indication for differences between the six semantic categories. We also complemented this evidence with data of body-part ratings for both action- and abstract-related sentences. Exploiting the classic method of identifying a category of entities by means of the combination of different traits, we provide a tentative synthetic table summarizing the main results of the present study (Table 6). Based on this table, we suggest the possibility of describing a particular pattern of characteristics for each category of sentence, which will be the main focus of the remaining part of our discussion.

**Table 6:** Synthetic summary of the main results of the present study.

	Mouth scale	Hand scale	Foot scale	CNC	CA	FAM
**Ms**	+	–	–	– –	– –	–
**Em**	+	+	+	– –	– –	–
**Ma**	–	+	–	+/–	+/–	–
**Mo**	++	–	–	+	+	+
**Ha**	–	++	–	++	++	+
**Le**	–	–	++	++	++	+

### Action-related Sentences

With respect to action-related meanings, we found a specific involvement of the mouth, the hands or the legs in the actions referred to, respectively, by mouth-, hand-, and leg-related sentences. Indeed, the distinctiveness of these action-related sentences has been observed in previous behavioral [26], and neuroimaging studies [28]–[30], and it is in general agreement with embodied cognition accounts [32], [34], [77], [90]–[91] highlighting the relevance of specific motor information for the semantic representation of action-related sentences. Here we completed the characterization of action-related sentences by ratings on concreteness, context availability and familiarity. In particular, we showed that mouth-related sentences were similar as far as familiarity is concerned, but were otherwise considered as being less concrete than hand- and leg-related sentences and less easily connected to a specific context than hand-related sentences, while still receiving higher concreteness and context availability scores than abstract-related meanings. Sentences with the lowest concreteness median scores (<6) were: *She mimes a face*; *She twists her lips*; *She tastes the wine*; *She savors the food*; *She relishes the champagne*.

The two sentences *She mimes a face* and *She twists her lips* can be considered as referring to non-verbal oro-facial communicative actions (verbal communicative actions were intentionally excluded from the present stimulus set), and thus considered of a more symbolic (i.e., “abstract”) kind than the remainder group of mouth-related sentences, in which an oro-facial motor involvement was generally coupled to a physical object to be ingested (e.g., *She bites the sandwich*; *She crunches the fruit*; *She swallows the pill*). In turn, the three sentences, *She tastes the wine*, *She savors the food*, and *She relishes the champagne*, albeit also referring to ingestive actions, were arguably associated with a somewhat peculiar function of “pleasure”, rather than strictly of “nourishment”. This more hedonistic function may be associated to increased sensory rather than solely motor attributes, thus maybe explaining the relatively lower concreteness scores. These data may suggest that the function of an action might be a component of its conceptual-semantic representation. Indeed functional knowledge is considered part of the information constituting the representation of object concepts, including knowledge about objects' function and more abstract propositional properties [80]. Neuropsychological and neuroimaging studies provided data showing how object concepts are represented in the brain as distributed networks including areas preferentially involved in the processing of sensory or functional knowledge [92]–[93]. The hypothesis might be tested and further extended to the other domains of action-related meanings in future research, by operationalizing the type of information available in processing action concepts. In any case, differences on concreteness and context availability between mouth- vs. hand- and foot-related sentences reveal that, even within the well-defined domain of concrete, action-related meanings, subtle differences between different categories can be identified that might be more deeply investigated in future studies.

### Mathematics-related Sentences

Mathematics-related sentences were judged as significantly engaging the hands more than the mouth and the legs. From a linguistic perspective, it is worth noting that there exist some Amazonian languages (such as Mundurukú) that lack words for numbers beyond 5 and use a broad variety of expressions such as “more than one hand”, “two hands”, “some toes”, “all the fingers of the hands” for referring to quantities greater than 5 [94]. Several lines of evidence indeed posit in favor of a possible relationship between finger counting and number processing, with number considered as a special kind of abstract concept [47]. Finger counting is a basic numerical learning strategy that develops spontaneously in infancy [95], supporting and preceding the acquisition of more advanced mathematical achievements [96]. Recent findings suggest that even in adults, finger counting patterns modulate arithmetic performance [97]. An increase in amplitude of motor-evoked potentials was found for the right hand muscles of subjects performing a visual parity judgment task on Arabic numerals [98], and on numbers and letters [99]. Recently, in a functional magnetic resonance imaging experiment, a signal increase was observed in the hemisphere contralateral to the hand used for counting when low numerosity numbers were presented, despite the absence of overt hand movement [100]. Our results extend such evidence in showing that hand-related semantic features can be identified at the semantic level in mathematics-related sentences. These results can be interpreted in the light of embodiment accounts, with the hand-related motor information as one of the possible modalities relevant for mathematics-related meaning.

Moreover, mathematics-related sentences appeared to be more concrete and more easily associated to a specific context than emotion- and mental state-related meanings, but lower in concreteness and context availability than action-related meanings. Interestingly, Dehaene and colleagues [101] proposed that internal representations of language-specific number words have a special role in mathematical thought: the use of number words (e.g., ‘ninety-eight’) is connected to the appreciation that each such number word names a distinct quantity (98-ness). Complementing the more basic biological capacities of individuating small quantities (such as, ‘1-ness’, ‘2-ness’, ‘3-ness’ and ‘more-than-that-ness’) and approximating magnitudes (for example, discriminating arrays of 8 dots from arrays of 16, but not more closely matched arrays) with the ability to use number words, humans can benefit of a simple and flexible method to think about an unlimited set of exact quantities. Speakers of Amazonian languages which do not have words for representing exact quantities rely on analogue magnitude estimation for estimating large quantities [102]. This may also occur in numerical-savvy English speakers when they are prevented from using linguistic resources by means of verbal interference tasks [103]–[104]. Although we didn’t use number words, but sentences describing mathematical operations, we might interpret the degree of concreteness and context availability as reflecting the fact that processing mathematics-related meanings may lead to the construction of quantities, which can easily be associated to contextualized concrete entities.

In sum, a strict classification of mathematics-related concepts as either concrete or abstract doesn’t seem to be appropriate. In this sense, mathematics-related concepts may constitute a case study of hybrid embodiment across the abstract and concrete domains, with a grounding in both abstract, reasoning mental processes and concrete, sensory-motor finger representations.

### Mental State- and Emotion-related Sentences

Even if emotion and mental-state meanings resulted similar with respect to concreteness, context availability and familiarity, they exhibited dissimilarities in the involvement of body parts, with emotion sentences more associated with mouth, hand and leg movements than mental-state and mathematics-related sentences. Recently, by means of event-related functional magnetic resonance imaging it has been shown that, in addition to a range of brain regions previously found to be active in emotion word processing, sensorimotor areas were also activated during the silent reading of abstract emotion words [37]. Specifically, signal increase was observed in the same areas entailed during the processing of face- and arm-related words, possibly suggesting that emotion words are associated to the involvement of specific districts of the body that are pivotal for displaying typical behaviors related to emotion. Importantly the emotional stimuli used in the experiment were words whose semantic meaning was either related to concrete or sensorimotor emotional actions (e.g., *frown*, *gnash*, *retch*) or not (e.g., *ail*, *rile*, *gloat*). Results were obtained for emotional words of both types, and further confirmed when only emotion stimuli not related to sensorimotor features were considered. By employing abstract emotion-related sentences (e.g., *She reveals the embarrassment*; *She mocks the disappointment*; *She experiences the excitement*) our results provide further evidence of an involvement of body-part representations (not limited to the mouth and the hands, but also including the legs) related to the semantics of emotion-related linguistic utterances.

It's worth noting that emotions and actions are supposed to be inter-related at anatomical and functional levels as follows [105]: i) the projections from the amygdala, which mediates emotional responses, to the brain stem may have influences on the generation of relatively simple, stereotypical motor responses and facial expressions; ii) the projections from the amygdala to the prefrontal cortex and the cingulate cortex may have influences on working memory and executive functions, which are crucial to higher-level planning and control of voluntary movements; iii) the emotional responses involve the autonomic and endocrine systems and provoke changes in the bodily states that may have some effects on action execution and control. It seems likely that emotion-related linguistic utterances evoke action-related features. According to embodied theories, emotion perception is linked to action simulation, since covert emotional states are often associated with overt motor behavior. Thus, observers can simulate and understand the observable emotional state of others by embodying their observable motor behavior [106]–[107]. In this view, emotion perception and action simulation are closely bounded together. Another line of research has suggested that emotional processing can trigger the motor system to prepare a motor act [108]–[110]. Defensive and approaching movements are triggered by unpleasant and pleasant cues, respectively [111]–[112]. Accordingly, we may speculate that high rating scores for the involvement of the legs in emotion related sentences may be due to defensive movement preparations elicited by emotion-related sentences. Still another possibility, however, is that motor components are tied to emotion-related linguistic utterances due to arousing semantic content, rather than as intrinsic embodied features.

In turn, mental-state meanings were specifically associated only to mouth movements. The mental-state related sentences that obtained the highest scores on the mouth scale (≥5) were: She memorizes the procedure; *She determines the fate*; *She discerns the opinion*; *She influences the choice*; *She pretends an interest*; *She assesses the views*. Within an embodied cognition framework, it is plausible that the meaning of these sentences integrates motor information about typical oro-facial activities that might be performed during a cognitive process, such as subvocal repetition during memorization processes or talking in order to take position or express personal opinions or views.

Although emotion and mental-state sentences seem to involve motor representations, they received very low concreteness and context availability scores. Abstract concepts are relational structures resulting from the integration of many different concepts in a situated conceptualization. For example, the concept of *to convince* integrates an agent, other people, an idea, communicative acts, possible changes in belief, talking with another, etc. [76]. The low context availability of emotion- and mental state-related sentences might reflect the difficulty in retrieving all such elements for the representation of the entire situated conceptualization. The body-part involvement can be considered as one of the dimensions of a relational structure that can dynamically become more or less relevant depending on the context.

### Conclusions

Altogether, the present study provided a fine-grained characterization of abstract meanings at the psycholinguistic level. We discussed the characterization of abstract-related categories especially in the light of recent proposals in the embodied cognition literature, suggesting that other theoretical accounts do not seem to explain within-domain meaning differences. These results are consistent with previous studies showing the distinctiveness of emotion-related concepts in terms of rating measures and neural underpinnings, and add important clues toward the possibility of identifying mathematics-related sentences as characterized by specific features within an hybrid abstract-concrete domain. Further research is necessary in order to investigate other important features related to abstract meanings. For example, in line with the traditional approach used by Russell [113] concerning emotion, investigating valence and arousal of linguistic utterances may reveal that these dimensions could differently mark emotion-related meanings.

In conclusion, these data inform future studies aimed at investigating the nature of different categories of concepts, indicating, for example, that also in the representation of abstract meanings sensory-motor maps may be significantly involved. Specifically, the ratings collected allow for a quantification of different profile of characteristics for action and abstract concepts, thus enabling the parametric manipulation of these characteristics in future research.
